# Practical management in Wolcott‐Rallison syndrome with associated hypothyroidism, neutropenia, and recurrent liver failure: A case report

**DOI:** 10.1002/ccr3.2168

**Published:** 2019-05-01

**Authors:** Markus Lundgren, Elisa De Franco, Henrik Arnell, Björn Fischler

**Affiliations:** ^1^ Department of Clinical Sciences, CRC Malmö Lund University Malmö Sweden; ^2^ Department of Pediatrics Kristianstad Central Hospital Kristianstad Sweden; ^3^ Institute of Biomedical & Clinical Science University of Exeter Exeter UK; ^4^ Department of Pediatric Gastroenterology, Hepatology and Nutrition Karolinska University Hospital Stockholm Sweden; ^5^ Division of Pediatrics CLINTEC, Karolinska Institutet Stockholm Sweden

**Keywords:** acute liver failure, monogenic diabetes, neonatal diabetes, pediatrics, skeletal dysplasia, Wolcott‐Rallison syndrome

## Abstract

Wolcott‐Rallison syndrome is a rare genetic syndrome of neonatal diabetes, liver failure, and growth retardation. We present a case with a *EIF2AK3* p.(Arg902Ter) mutation, additionally complicated by hypothyroidism, impaired renal function, and exocrine pancreas insufficiency, focusing on clinical management. For its optimization, thorough care of multiple organ systems is needed.

## 1 INTRODUCTION

Wolcott‐Rallison syndrome (WRS, OMIM 226980), first described in 1972,^1^ is a rare form of neonatal diabetes caused by homozygous or compound heterozygous mutations in the *EIF2AK3* gene. The gene encodes the enzyme protein kinase R‐like endoplasmic reticulum kinase (PERK) which is vital for the ability of cells to handle endoplasmic reticulum (ER) stress.^2^ Loss of function in PERK leads to apoptosis in situations of increased ER stress, resulting in permanent diabetes mellitus, multiple epiphyseal dysplasia, growth failure, and recurrent liver failure.^3^, ^4^ Several other findings have been described in association with WRS, among them primary hypothyroidism, exocrine pancreas insufficiency, renal failure, neutropenia, anemia, central nervous system abnormalities, and developmental delay.^5^, ^6^, ^7^, ^8^

Neonatal diabetes is, in most cases, the presenting symptom. However, the phenotype is variable both with regard to organs involved and clinical severity. To this day, data on fewer than 100 children have been published, with most clinical centers only caring for a single patient. Morbidity, as well as mortality, is high among children with WRS.^7^, ^9^

Here, we present the first published Swedish case of WRS complicated by recurrent severe liver dysfunction, neutropenia, and kidney dysfunction as well as primary hypothyroidism and skeletal dysplasia. Our primary aim was to give a practical view and experience on management of a patient with WRS at a secondary pediatric center in southern Sweden.

## 2 CASE REPORT

The patient was born at 39 + 6 weeks with a birthweight of 2675g (−1.9 SDS) and birth length 47 cm (−1.6 SD) after an uncomplicated pregnancy. She was the first child of nonconsanguineous, healthy, parents of Albanian origin. There was no family history of diabetes or early deaths in the family. The patient was first admitted at 5 weeks of age with fever consistent with viral infection. At admission, she was hyperglycemic (blood glucose 41 mmol/L) but not in diabetes ketoacidosis (pH 7.35) and with an HbA1c of 32 mmol/mol (5.5%). After initial treatment with intravenous insulin (starting dose 0.025 U/(kg × h), she was put on an insulin pump (Medtronic Minimed^®^) with initial insulin requirement at discharge of 1.3 U/(kg × d). Bolus doses of regular 100 U/mL rapid‐acting insulin were determined using carbohydrate counting, where insulin to carbohydrate ratios were calculated by determining the blood glucose response to insulin and estimating the carbohydrate ratios accordingly. Blood glucose measurements in infancy were performed with finger pricks due to parental opposition to using continuous glucose (CGM) measurements. An ultrasound of the abdomen showed a pancreas and liver of normal size and appearance. Diabetes‐related autoantibodies (IAA, GADA, IA‐2A, and ZnT8A) were negative, and HLA genotyping revealed a genotype of DQA1*01‐DQB1*06, DQA1*05‐DQB1*0301 (HLA DQ6/7). Genetic testing for neonatal diabetes was performed by the Exeter Molecular Genetics laboratory using the previously published pipeline.^10^ Direct sequencing of the KCNJ11, INS, and ABCC8 genes was performed by PCR followed by Sanger sequencing, but no likely pathogenic variant was identified. Testing of all other known neonatal diabetes genes, including EIF2AK3 (AF110146.1), was performed using a custom designed targeted next‐generation sequencing panel test, as previously described.^11^ The bioinformatics tools SIFT, PolyPhen‐2 and Align GVGD, were accessed through the ALAMUT Visual software version 2.7.1 (Interactive Biosoftware) to predict the effect of novel variants. The EIF2AK3 variant was confirmed by PCR and Sanger sequencing of exon 13 as a homozygous nonsense mutation (p.(Arg902Ter), c.2704C > T), previously described in WRS.^9^ Both parents were confirmed to be heterozygous carriers of the same mutation.

**Figure 1:**
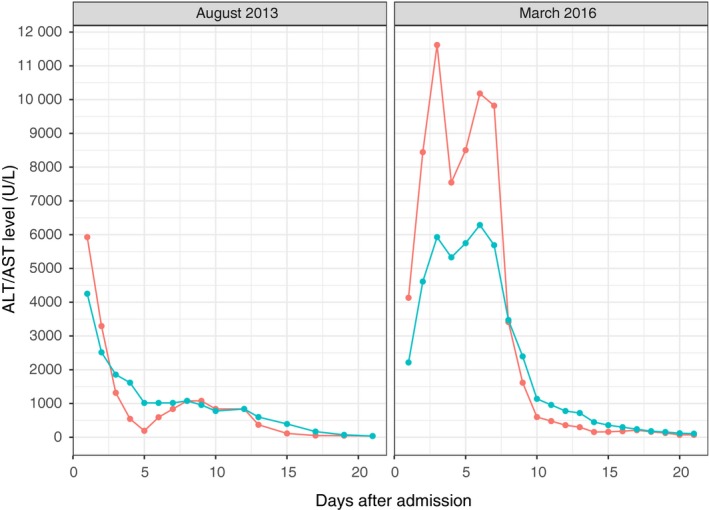
Liver enzymes (AST (blue) and ALT (red)) during the patient's first (left panel) and most severe (right panel) exacerbations of liver dysfunction

At 3 months of age, following the first vaccination against diphtheria, tetanus, and pertussis, she presented at the emergency room with lethargy, fever, hyperglycemia, and dehydration. Initial laboratory analysis revealed a blood glucose level of 13.5 mmol/L, no acidosis (pH 7.36), increased lactate (6.1 mmol/L), and markedly increased liver function tests (LFTs) with AST 5900 U/L (ref. 20‐60 U/L) and ALT 4270 U/L (ref. 6‐50 U/L), ALP 444 U/L (ref. 110‐320 U/L), total bilirubin of 30 µmol/L (ref. 3.4‐17 µmol/L), conjugated bilirubin 27 µmol/L, and a prothrombin time measured as INR of 1.5 (ref. 0.8‐1.2). Establishing venous access was difficult, and a central venous catheter was ultimately needed, which was later replaced by a subcutaneous venous port. During hospitalization, episodes of absences and suspected seizures ensued which lead to transfer to a tertiary regional center. An epilepsy work‐up was performed with a normal brain CT, normal EEG, and slightly increased protein levels in cerebrospinal fluid, consistent with blood‐brain barrier damage. Eventually, she also developed neutropenia (0.7 × 10^9^/L) which spontaneously normalized. The initial exacerbation of severe liver dysfunction subsided after 10 days with complete biochemical resolution including LFTs and neutrophils (Figure 1).

Endocrine pancreatic function was assessed at first admission with a nonfasting c‐peptide level of 0.12 nmol/L (ref. for fasting sample: 0.37‐1.5 nmol/L). At repeat measurements, c‐peptide was below the threshold of the analysis method. Fecal elastase, first analyzed after the age of one year, was repeatedly low around 70 µg/g (ref. >200 µg/g). Thyroid‐stimulating hormone was consistently elevated from one year of age (7‐11 mIE/L), with no thyroid autoantibodies (TPO and TRAK) present, and levothyroxine therapy was initiated.

During the following 3.5 years, the patient suffered an additional six exacerbations requiring in‐patient care, all including severely elevated AST, ALT, and INR, and often neutropenia and compromised renal function. The most severe exacerbation occurred at three years of age following an uncomplicated viral infection. The patient was generally unwell and admitted with elevated LFTs (AST 4100 U/L; ALT 2215 U/L; INR normal on admission) (Figure 1). She had no ketoacidosis but was lethargic with decreased consciousness. During the coming days, she developed acute liver failure and was transferred to a pediatric hepatology and liver transplant unit to prepare for emergency liver transplant in case of fulminant progressive liver failure. At admission, at the tertiary center the patient displayed signs of grade 3 hepatic encephalopathy (somnolence, confusion and gross disorientation). Liver enzymes reached a maximum ALT of 11.600 U/L, AST of 5.928 U/L with a maximum INR of 3.8 with a concurrent ammonia level of 91 µmol/L (ref. 11‐51 µmol/L). Her general well‐being and liver function rapidly improved after 5 days of supportive therapy with antibiotics, lactulose, phytomenadione, acetylcysteine, rifaximin, and nutritional supplementation. However, as liver function improved, renal function deteriorated with increased serum levels of creatinine (245 µmol/L, ref. 22‐53 µmol/L), blood urea nitrogen (BUN; 17 mmol/L), and eventually anuria. Continuous hemodialysis was initiated and continued for 8 days. The episode of renal failure was deemed likely to be caused by the exacerbation of WRS. Liver function tests subsequently returned to normal although renal function remained slightly affected.

Two exacerbations of liver dysfunction and neutropenia temporally correlated with vaccination against diphtheria, tetanus, and pertussis (DTaP), administered at three and five months of age, respectively. After this, no other vaccinations were administered to the patient. The patient underwent anesthesia on several occasions, with no adverse events recorded. Sevoflurane, propofol, midazolam, rocuronium, atracurium, morphine, clonidine, and remifentanil were used, in standard dosing per kg. For pain and fever management, acetaminophen (paracetamol) was used, especially with intercurrent liver enzyme elevation. The dosing was reduced at times of decreased liver synthetic function, as indicated by rising INR.

**Figure 2:**
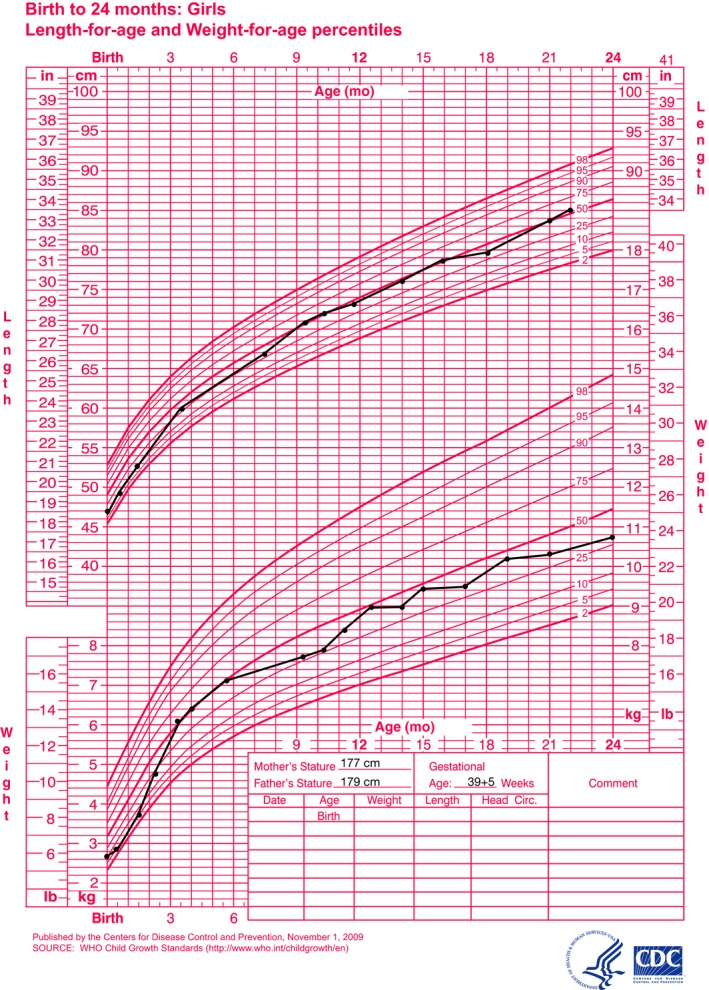
Weight and length for age growth chart for 0‐24 months of age

During the patients first years of life, the growth rate was normal, with no significant change in length and weight SDS. After an initial catch‐up following initiation of insulin therapy, she displayed a normal growth trajectory (Figure 2). Initial x‐ray examination, at age 16 months, did not show any signs of epiphyseal dysplasia or osteopenia. However, when repeated at age 31 months clear signs of epiphyseal dysplasia were evident in metacarpals, carpals, radius, femur, tibia as well as possible metaphyseal dysplasia in carpal and metacarpal bones. At age 2.5 years, her length was 88.5 cm (−1.1 SDS; 14th percentile) and weight 13 kg (−0.64 SDS; 26th percentile). The patient reached her developmental milestones and showed no clear signs of developmental delay.

At three years of age, the patient was referred to a tertiary liver center for consideration for elective liver transplantation.

## 3 DISCUSSION

WRS is a rare autosomal recessive disorder with diverse phenotypic traits. Neonatal diabetes and epiphyseal dysplasia are considered consistent canonical features and can be accompanied by a variety of other manifestations. Dysfunction in several organ systems has been associated with the syndrome, and the pathogenesis is deemed to be caused by dysfunction of the PERK protein. This molecular defect leads to a decreased ability of the ER to handle protein folding during times of increased protein synthesis caused by increased demands and stress. The accumulation of unfolded proteins in the ER results in an apoptosis signal and cell death leading to organ damage.^2^, ^12^

Our patient is homozygous for a nonsense mutation in exon 13 of the *EIF2AK3* gene (c.2704C > T, p.(Arg902Ter)) first described in 2009. To our knowledge, this is the fifth published case of WRS with the same mutation in children of Albanian descent.^13^ Unfortunately, clinical limited clinical data are only published on two of these children. This additional published case further strengthens the hypothesis of a founder effect in this relatively small population. She was diagnosed with neonatal diabetes at five weeks of age. Glycemic control was complicated mostly by problems related to a diet heavy on infant formula, gruel, and porridge. She had no noticeable problems with hypoglycemia, aside from what is expected in insulin‐dependent diabetes. This contrasts with previous reports of problematic hyper‐ and hypoglycemia associated with WRS.^6^

The first episode of liver failure followed a routine vaccination with DTaP at three months of age. Whether the therapeutic attempts influenced the clinical course of this and the later episodes, or whether it was self‐limiting, remains unclear. At the present time, there is no published data suggesting treatment choices in WRS which further complicates treatment choices. That special care needs to be taken in conjunction with medications in patients with WRS has been reported previously (1,14). In our patient, however, only vaccinations were temporally associated with disease exacerbations, whereas all other medications were used without unexpected events. This may reflect the heterogeneity of the syndrome but may also highlight that the timing and setting of administration influence the potential side effects. In addition to liver dysfunction, a repeated pattern of moderately elevated serum levels of creatinine was found during exacerbations with a maximum reached at age three years where one exacerbation subsequently resulted in renal failure and the need for dialysis. Impaired renal function has been associated with WRS in previous reports,^9^ although in contrast to ours described as a transient.

She was also diagnosed with primary hypothyroidism. This is in line with earlier reports proposing primary hypothyroidism as part of the phenotype in some patients with WRS^14^, ^15^ and especially with the AF110146.1_p.(Arg902Ter) mutation present in our patient and in previously published cases.^13^ Growth and developmental milestones were unremarkable in during the first 2.5 years. This is in contrast to previously published case reports where several patients have displayed faltering growth from approximately one year of age.^7^, ^16^ Further follow‐up will clarify whether this positive trend will remain or whether faltering growth will ensue with time.

## 4 CONCLUSION

Wolcott‐Rallison syndrome is an extremely rare and severe syndrome with highly variable clinical presentation where most patients are the only patient at their respective centers. Hence, each new patient is a challenge to the responsible physician, requiring a multi‐disciplinary approach. Careful and consistent evaluation of glycemic control, as well as renal, thyroid and pancreatic function and growth is needed. The patients are prone to repeated episodes of acute liver failure which may increase in severity with time with the subsequent risk of developing life‐threatening fulminant liver failure. Liver transplantation is an emerging concept, both as rescue treatment and as elective treatment, for patients with WRS as well as other metabolic diseases and may improve clinical outcomes.^17^, ^18^

## CONFLICT OF INTEREST

None declared.

## AUTHOR CONTRIBUTION

ML collected the data, was the responsible clinician and wrote the manuscript. HA, BJ, and EDF substantially contributed to the interpretation of the genetic data and critically revised the manuscript for important intellectual content. ML is responsible for the integrity of the work as a whole.

## STATEMENT OF INFORMED CONSENT

Informed consent was obtained from both parents of the patient prior to publication.
